# Evaluating the Clinical Utility of Robotic Systems in Plastic and Reconstructive Surgery: A Systematic Review

**DOI:** 10.3390/s25103238

**Published:** 2025-05-21

**Authors:** Ishith Seth, Kaiyang Lim, Edmond Chang, Warren M. Rozen, Sally Kiu-Huen Ng

**Affiliations:** 1 Department of Plastic and Reconstructive Surgery, Austin Health, Heidelberg, VIC 3084, Australiasally.ng@austin.org.au (S.K.-H.N.); 2 Faculty of Medicine and Surgery, Peninsula Clinical School, Monash University, Frankston, VIC 3199, Australia; 3 Department of Plastic and Reconstructive Surgery, Peninsula Health, Frankston, VIC 3199, Australia

**Keywords:** robotic, plastic surgery, reconstructive surgery, microsurgery, systematic review

## Abstract

**Background**: Robotic surgical systems offer enhanced precision, motion scaling, tremor filtration, and visualization, making them highly suitable for the complex anatomical demands of plastic and reconstructive surgery. While widely implemented in other specialties, their integration in plastic surgery remains limited. This systematic review evaluates the clinical applications, outcomes, and limitations of robotic-assisted techniques in plastic and reconstructive procedures. **Methods**: Following PRISMA guidelines, a systematic search was conducted across PubMed, Embase, Scopus, and Web of Science for studies published between January 1980 and March 2025. Clinical studies reporting robotic applications in plastic surgery were included, while cadaveric, animal, and non-English studies were excluded. Data extraction and quality assessment were performed using Covidence and validated tools including the CARE checklist, NOS, GRADE, and SYRCLE. A total of 1428 studies were screened, and 31 met the inclusion criteria. **Results**: Robotic systems were primarily applied in microsurgery (*n = 16*), breast reconstruction (*n = 8*), and craniofacial/aesthetic surgery (*n = 7*). Common platforms included the Symani Surgical System, Da Vinci systems, and ARTAS. Robotic-assisted approaches improved precision, aesthetic outcomes, flap survival, and patient satisfaction, particularly in procedures involving lymphaticovenous anastomosis and nipple-sparing mastectomy. However, challenges included steep learning curves, longer operative times, high equipment costs, and the lack of haptic feedback. Quality assessment rated all studies as moderate. **Conclusions**: Robotic-assisted surgery demonstrates considerable potential in enhancing plastic and reconstructive outcomes. As systems become more compact, cost-effective, and integrated with AI and biomimetic technologies, their broader adoption is anticipated. Further high-quality studies are needed to optimize these systems and support widespread clinical implementation.

## 1. Introduction

Plastic and reconstructive surgery is a highly specialized field that demands exceptional precision, dexterity, and adaptability to restore form and function [1]. Technological innovations have played a crucial role in enhancing surgical outcomes, particularly in procedures such as microsurgery, craniofacial reconstruction, and breast reconstruction, due to their intricate nature [2]. Among these advancements, robotic-assisted surgery has emerged as a transformative tool, offering improved visualization, motion scaling, and tremor reduction to address the physical and cognitive challenges surgeons face [3].

Robotic-assisted surgery has revolutionized microsurgical practices by offering unparalleled precision, motion control, and the ability to overcome human physiological limitations, such as tremors [4]. The Symani Surgical System^®^ represents a state-of-the-art robotic platform engineered explicitly for microsurgery [5]. By incorporating advanced features such as motion scaling, tremor filtration, and ergonomic enhancements, the system enables surgeons to perform intricate procedures with greater accuracy and stability. These features are particularly advantageous for super microsurgical applications, such as reconstructive surgery and hand trauma care, where precision is paramount, and the operating field often involves vessels as small as 0.6 mm in diameter [6].

Adopting robotic platforms, such as the Symani Surgical System, can redefine the scope of microsurgical interventions by increasing surgical precision and reducing tissue trauma [6]. However, integration into clinical practice depends heavily on understanding the associated learning curve, particularly for surgeons accustomed to manual techniques. The initial training phase poses unique challenges, including adapting to the absence of tactile feedback, mastering navigation within a virtual workspace, and adjusting to the robotic system’s distinct suture-tying methods [7]. These barriers must be addressed to ensure the system’s successful implementation and widespread adoption.

Evidence from the literature highlights the promising clinical efficacy of the Symani Surgical System^®^. Mori et al. demonstrated a 100% success rate in free flap reconstructions performed with Symani, emphasizing its utility in supermicrosurgical procedures [7]. Dastagir et al. reported comparable patency rates between robotic and manual anastomoses, with robotic performance approaching manual efficiency after 10 cases [5]. Other studies corroborate these findings, documenting significant improvements in operative times and surgical outcomes as surgeons become proficient with the system [8]. These results highlight the potential of the Symani Surgical System to achieve outcomes equivalent or superior to those of conventional techniques in various clinical settings [8].

In addition to the Symani Surgical System^®^, several other robotic platforms have contributed to the advancement of plastic and reconstructive surgery. The Da Vinci Surgical System is the most widely adopted robotic platform globally, offering articulated arms with wrist-like motion, 3D high-definition imaging, and tremor filtration. The Craniofacial-Plastic Surgical Robot (CPSR-I) system is a specialized platform designed for craniofacial applications, integrating force feedback and automated osteotomy to enhance bony contouring accuracy and reduce revision rates [9]. The ARTAS Robotic System, primarily used in aesthetic surgery, leverages stereoscopic imaging and intelligent algorithms to perform follicular unit extraction with high precision and minimal trauma, improving outcomes in hair transplantation [9]. These systems collectively illustrate robotic platforms’ diverse and expanding role in addressing the anatomical complexity and precision demands of modern reconstructive surgery.

Despite its advantages, the robotic system has its limitations. Challenges identified in previous studies include inadequate instrument grip strength, inefficiencies in manipulating thin sutures, and limited range of motion in specific scenarios. The absence of tactile feedback has been a recurring issue, with some surgeons developing a “see-feel” technique to compensate for the lack of physical sensation [10]. Additionally, operational inefficiencies, such as controller misalignment within the virtual workspace, have been reported as a source of delays [10]. The adoption of robotic systems in plastic and reconstructive surgery faces several challenges. Prolonged operative times, high costs, limited haptic feedback, and the steep learning curve associated with robotic-assisted procedures remain significant barriers to widespread implementation. Furthermore, while robotic platforms enhance surgical precision and reduce surgeon fatigue, their effectiveness in improving long-term clinical outcomes and patient satisfaction warrants further investigation.

This systematic review aims to critically evaluate the current applications of robotic surgical systems in plastic and reconstructive surgery. By synthesizing available evidence, we seek to assess their impact on clinical outcomes, explore the advantages and limitations of robotic-assisted techniques, and identify potential areas for future research and innovation. Understanding the role of robotics in this evolving field will provide valuable insights into optimizing surgical techniques and expanding the scope of reconstructive procedures.

## 2. Materials and Methods

### 2.1. Study Design

This systematic review was conducted in accordance with the Preferred Reporting Items for Systematic Reviews and Meta-Analyses (PRISMA) guidelines to evaluate the use of robotic surgery in plastic and reconstructive procedures. The review protocol was registered in PROSPERO. A comprehensive literature search was conducted across PubMed, Embase, Scopus, and Web of Science, encompassing studies published from 1 January 1980 to 10 March 2025. The search strategy incorporated Boolean operators and controlled vocabulary to refine the results, with primary keywords including “robotic surgery” OR “surgical robots” AND “plastic surgery” OR “reconstructive surgery”. Additionally, specific robotic platforms, such as the Da Vinci and Symani robotic systems, were included to ensure comprehensive coverage of the relevant literature. All the studies’ reference lists were also screened to identify additional relevant articles.

### 2.2. Inclusion and Exclusion Criteria

Studies were included if they were peer-reviewed clinical investigations, such as randomized controlled trials (RCTs), cohort studies, prospective studies, and case series, that reported the application, surgical outcomes, and challenges of robotic-assisted surgery in plastic and reconstructive procedures. Only studies published in English were included. Exclusion criteria included cadaveric and animal studies, non-English publications, case reports, review articles, conference abstracts, and studies unrelated to robotic surgery in plastic and reconstructive surgery. Data extraction was conducted using Covidence (2025) to facilitate systematic screening. Two independent reviewers (IS and KL) assessed study eligibility, and a third reviewer resolved any discrepancies. Extracted data included study type, robotic system used, procedural details, surgical outcomes, complication rates, operative time, and technical limitations. The results were systematically categorized into key domains, including robotic applications in microsurgery, breast reconstruction, craniofacial surgery, and other reconstructive procedures.

### 2.3. Risk of Bias

The risk of bias was evaluated using the Newcastle–Ottawa Scale (NOS) for observational studies, CARE checklist, SYRCLE and GRADE assessment for case series, and the Cochrane Risk of Bias (RoB 2) tool for RCTs to assess the quality and reliability of included studies. These tools evaluated factors such as study design, selection bias, blinding, outcome assessment, and reporting quality. Studies were rated as having a low, moderate, or high risk of bias, and those with substantial methodological limitations were noted but not excluded unless they failed to meet fundamental inclusion criteria. Given the heterogeneity in study design and outcome measures, a meta-analysis could not be conducted to summarize findings; hence, quantitative pooling or meta-analysis was considered if sufficient comparable data were available.

## 3. Results

### Literature Results

**Figure 1:**
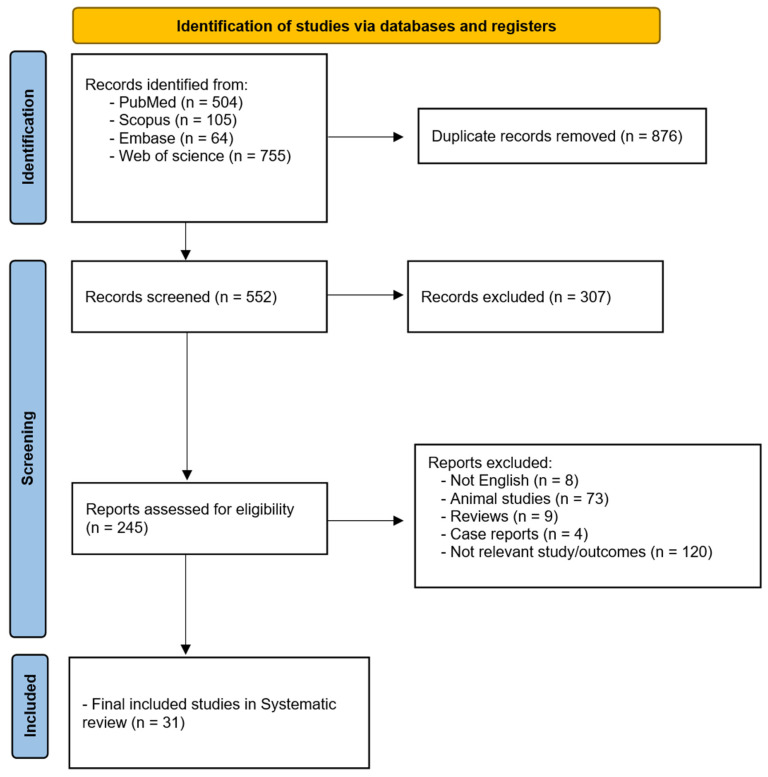
PRISMA diagram of study selection.

A total of 1428 studies were identified using database searches. After removing duplicates and screening for relevance, 31 studies met the inclusion criteria. Figure 1 illustrates the PRISMA diagram of the study selection. These studies, published between 1 January 1980 and 10 March 2025, examined the use of robotic surgery in plastic and reconstructive procedures, with a focus on microsurgery, breast reconstruction, and facial reconstruction.

Microsurgery emerged as the most extensively studied area, with 16 studies exploring robotic-assisted nerve repair, vascular anastomosis, and hand microsurgery. Eight studies focused on breast reconstruction, including robotic DIEP flap harvesting, latissimus dorsi flap harvesting, and nipple-sparing mastectomy (NSM). The remaining nine studies addressed head and neck reconstruction, craniofacial contouring, upper facial rejuvenation, and hair transplantation.

Robotic platforms used included the Symani Surgical System, various Da Vinci systems, and the ARTAS robotic system. Several studies highlighted the role of robotics in advancing complex reconstructive techniques, such as simultaneous flap harvesting and microsurgical anastomosis. These innovations have proven especially valuable in managing intricate reconstructions, including oncologic defects and severe burn scars. A comprehensive overview of study characteristics is presented in Table 1. A quantitative meta-analysis could not be performed due to significant heterogeneity between the studies.

The quality assessment revealed no studies rated as high quality (Table 2). Most included studies were assessed as moderate quality, primarily due to methodological limitations such as small sample sizes, lack of control groups, retrospective or case series designs, and limited follow-up data. While several studies employed validated assessment tools, such as the CARE Checklist for case series and the Newcastle–Ottawa Scale for cohort studies, downgrades were frequently applied due to the risk of bias and incomplete reporting of outcomes. Preclinical studies, assessed using the SYRCLE risk of bias tool, also demonstrated moderate risk due to limited surgeon variability and translation challenges to clinical settings. Despite these limitations, the consistent use of robotic systems across diverse procedures and settings supports the feasibility and safety of robotic-assisted plastic and reconstructive surgery. However, further high-quality, prospective, and comparative studies are warranted to strengthen the evidence base.

Robotic systems have shown transformative impact across various subspecialties within plastic and reconstructive surgery. In microsurgery, the Symani Surgical System was the most utilized platform, facilitating complex procedures such as lymphaticovenous anastomoses (LVA) and peripheral nerve coaptation. Its features include motion scaling, tremor elimination, NanoWrists with up to seven degrees of freedom, and high-definition 3D visualization, enabling the safe handling of submillimeter vessels, particularly in super microsurgical and hand trauma scenarios [24,32,34]. Studies have also reported successful multi-nerve coaptations, resulting in improved functional outcomes and ergonomic benefits.

In breast reconstruction, robotic systems were frequently applied to DIEP flap harvesting, latissimus dorsi flap procedures, and nipple-sparing mastectomy. Robotic-assisted approaches allowed for more precise pedicle dissection and reduced fascial trauma, although operative times remained longer in early cases. However, Jung et al. [33] demonstrated that operative efficiency improved with experience, as the mean operative time decreased from 378.1 to 334.2 min after 21 cases. Moreover, robotic-assisted flap harvesting demonstrated better preservation of abdominal wall integrity and reduced rates of postoperative hernia and pain [22,35,36].

Robotic technology has also expanded into craniofacial and aesthetic surgery, including facial contouring and upper face rejuvenation. The Da Vinci system offered enhanced surgical precision and ergonomics, particularly in subperiosteal dissections. In hair transplantation, the ARTAS robotic system employed intelligent algorithms and stereoscopic imaging to optimize graft placement, improve consistency, and minimize follicular damage, resulting in high patient and surgeon satisfaction scores [18].

These robotic systems are grounded in mechanotactic principles, replicating natural human dexterity and visual perception. The Symani system, with up to 20× motion scaling and tremor filtering, enabled exact maneuvers in deep and confined anatomical spaces [25,27]. Similarly, Da Vinci platforms mimic wrist articulation and binocular vision, enhancing maneuverability and depth perception. The ARTAS system applied nature-inspired logic to replicate natural hair growth patterns, increasing the accuracy and efficiency of follicular unit placement [18]. The Da Vinci Surgical System is FDA approved for various general, gynecologic, and urologic procedures, and its use in breast and head and neck surgeries is considered off-label but widely practiced. The Symani Surgical System received CE Mark approval in Europe and recently provisional approval in Australia for microsurgery applications, but it has not yet been FDA approved. The ARTAS Robotic System is FDA cleared specifically for hair transplantation procedures. In contrast, the Craniofacial-Plastic Surgical Robot remains experimental and has not received formal regulatory approval for clinical use. Clinicians should be aware of these distinctions, especially when considering device use outside approved indications.

Clinically, robotic-assisted procedures consistently demonstrated improved precision, lower complication rates, and higher patient satisfaction. In NSM, studies reported significantly reduced rates of skin necrosis with robotic approaches—2% versus 8% in conventional surgery [30]—and improved patient-reported outcomes, including higher BREAST-Q scores for sexual and psychosocial well-being [14,30]. Postoperative pain was also reduced in robotic breast surgery groups, with Lee et al. [35] reporting significantly lower pain scores in the first 24 h.

In microsurgery, platforms like Symani enabled complex reconstructions such as multi-vessel anastomoses in free flaps, with studies reporting 100% anastomotic patency and no postoperative complications [24]. Operative times decreased with experience, as shown by Barbon et al. [39], and LVA procedures resulted in substantial limb volume reduction without complications [21]. These findings reinforce the system’s increasing reliability with growing surgical experience.

In aesthetic and craniofacial surgery, robotic systems provided enhanced control over soft tissues and bony structures, reducing swelling and improving contouring precision. Force feedback mechanisms have further improved accuracy in delicate tasks, such as genioplasty, thereby minimizing the need for revision surgery [17]. Additionally, Lai et al. [13] demonstrated 100% flap survival in head and neck reconstruction using robotic-assisted microsurgery, with precise vascular suturing and minimal complications.

## 4. Discussion

Robotic-assisted surgery is increasingly being adopted across various surgical specialties, with growing applicability in plastic and reconstructive surgery. The advantages of robotic platforms, including motion scaling, tremor filtration, articulated instrumentation, and 3D high-definition visualization, have proven particularly valuable in procedures requiring fine precision, such as microsurgery, breast reconstruction, and craniofacial surgery (Table 3). These systems enhance operative dexterity, reduce surgeon fatigue, and enable less invasive approaches, improving aesthetic and functional outcomes.

In breast and reconstructive procedures, the da Vinci Surgical System has consistently improved precision, scar minimization, and postoperative satisfaction by mimicking human wrist movement and allowing refined dissection in confined spaces [13,15,16,19,20,31,35]. For instance, robotic-assisted nipple-sparing mastectomy has shown superior scar positioning and reduced skin necrosis rates [14,30]. At the same time, robotic DIEP flap harvesting resulted in preserved abdominal wall integrity and earlier drain removal [20]. However, limitations such as robotic arm collisions in constrained spaces, as reported by Chung et al. [10], underscore the need for improved operating room ergonomics and potentially larger surgical theatres.

Microsurgery was the most extensively explored field in this review, with the Symani Surgical System widely employed due to its capacity to perform high-precision tasks, such as lymphatic-venous anastomoses, free flap reconstruction, and peripheral nerve coaptation. With features such as motion scaling up to 20× and NanoWrist instruments with seven degrees of freedom, the Symani system enables the safe handling of vessels smaller than 0.3 mm, improving surgical accuracy, even in anatomically challenging sites [24,27,32]. Studies such as those by Struebing et al. and Besmens et al. reported 100% anastomotic patency rates and consistent improvements in operative efficiency over time.

The application of biomimetic principles extends beyond mechanical design. Recent developments in surface coatings mimic biological tissues to improve selective adhesion or reduce friction, while adaptive control algorithms refine movement in real-time, enhancing surgical precision and tissue safety. These innovations are particularly relevant in microsurgical tasks such as free flap dissection or vascular suturing, where tactile sensitivity and stability are paramount.

Emerging technologies also point toward the future miniaturization and softening of robotic platforms. “Soft robots”, inspired by organisms such as jellyfish and starfish, offer increased flexibility, constant pressure distribution, and adaptability to irregular anatomical surfaces—attributes that could be revolutionary in delicate tissue handling during reconstructive procedures [40]. Innovations such as gecko-inspired microadhesive structures may further improve robotic grasp on slippery or fragile tissue, reducing trauma and increasing control during grafting or suturing [41].

Artificial intelligence (AI) is poised to play a pivotal role in the next generation of robotic surgery. AI-driven systems are being developed to enhance preoperative planning, simulate operative strategies, and provide real-time intraoperative guidance [42,43,44,45]. Machine learning algorithms can identify tissue planes, highlight high-risk anatomical regions, and offer operative suggestions, thereby optimizing surgical flow and minimizing errors [42,43,44]. Augmented and virtual reality (AR/VR) technologies complement this by integrating imaging modalities (e.g., CT, MRI) with real-time simulation tools, allowing surgeons to visualize complex anatomy and rehearse procedures [45]. Lin et al. [17] demonstrated that AR-enhanced robotic-assisted genioplasty improved preoperative planning and intraoperative precision, showcasing the potential of integrated visual computing technologies in reconstructive surgery.

Several challenges we identified when using the Symani Surgical System^®^ (Medical Microinstruments, S.p.A, Calci, Pisa, Italy) were also reported in the literature. Perspectives on the lack of tactile feedback varied as widely in the literature as in this study. Struebing et al., for example, expressed that ‘see-feel’ was sufficient to replace tactile input across a range of free flap, lymphatic, and nerve reconstructions [14]. In contrast, Besmens et al. expressed that ‘see-feel’ was insufficient for gauging the correct tension for microvascular anastomoses [4]. A combination of individual surgeon preferences and case-specific demands likely contributes to this variance of opinions. Tolksdorf et al. encountered thin sutures slipping through the grip of robotic instruments, a phenomenon also observed in this study [15]. This issue is particularly pertinent, given that the Symani Surgical System’s key advantage is its motion scaling and tremor reduction technology, which facilitates surgery with thin sutures and small structures. It would be valuable to understand why other studies that used 10-0 and 11-0 sutures did not encounter instrument grip issues, whether related to certain suture materials or specific batches of robotic instruments [46]. Several improvements to robotic instrument design should be considered to enhance patient safety and reduce intraoperative complications. Enhancing the grip strength and precision of microsurgical instruments would improve the handling of delicate sutures and tissues, particularly in lymphatic and nerve procedures. Integrating haptic feedback or real-time force sensors could help overcome the lack of tactile sensation and reduce vessel or nerve injury. Additionally, miniaturizing robotic arms and improved angulation could facilitate access in confined anatomical spaces, while adaptive control systems could improve stability during fine movements.

Despite these advantages, several challenges remain. Nearly half of the studies reported longer operative times than conventional techniques, often due to setup, docking, and early-phase learning curves [13,14,20,30,33]. However, multiple studies also demonstrated significant reductions in operative duration with increasing surgical experience. For example, Jung et al. [33] observed a marked decrease in total operation time after 21 robotic-assisted NSM procedures, highlighting the importance of training and case volume in improving workflow efficiency. Several studies included in this review highlighted the steep learning curve associated with robotic-assisted procedures, particularly during the initial implementation phase. While a universally accepted quantitative threshold for proficiency is lacking, some studies reported noticeable improvements in operative times and efficiency after approximately 10 to 30 cases, depending on the procedure and robotic platform used [5,24,33]. For instance, Jung et al. [33] observed a significant reduction in total operative time after 21 cases of robotic-assisted nipple-sparing mastectomy. Similarly, Dastagir et al. [24] noted improved anastomosis times and ergonomic benefits with increasing experience. These findings suggest that consistent practice and procedural volume are critical to overcoming the learning curve and achieving surgical proficiency. Structured training programs, simulation-based practice, and mentorship models may facilitate skill acquisition and improve the safety and efficiency of robotic-assisted plastic surgery.

Furthermore, the high acquisition, maintenance, and training costs associated with robotic systems and spatial constraints in operating theatres were common limitations noted across studies. These economic and logistical barriers may restrict access to robotic surgery in lower-resource settings. Future research should include cost-effectiveness analyses and focus on developing more compact, transportable, affordable systems. Expanding access to robotic platforms could democratize their use, ensuring patients in regional or underserved areas also benefit from technological advances. While several studies noted the higher costs and longer operative times associated with robotic-assisted procedures, direct comparative studies on cost-effectiveness remain limited. Jeon et al. [16] and Tsai et al. [22] reported increased disposables and operative duration expenses. However, improvements in efficiency with experience, as shown by Jung et al. [33], suggest potential long-term gains. Some studies also noted enhanced cosmetic outcomes and reduced morbidity, which may offset costs, though formal economic evaluations are lacking. Further research is needed to assess the cost-effectiveness of robotic-assisted approaches relative to conventional methods.

## 5. Conclusions

This systematic review highlights robotic-assisted surgery’s emerging role and growing utility in plastic and reconstructive procedures. Robotic systems, particularly the Da Vinci Surgical System and the Symani Surgical System, have shown significant promise in enhancing surgical precision, reducing tissue trauma, and improving functional and aesthetic outcomes across various domains, including microsurgery, breast reconstruction, craniofacial surgery, and aesthetic procedures. In microsurgery, advanced motion scaling and tremor filtration has enabled the safe manipulation of submillimeter vessels and nerves, facilitating high patency rates and improved operative efficiency with experience. Similarly, in breast reconstruction, robotic-assisted nipple-sparing mastectomy and DIEP flap harvesting have demonstrated superior scar positioning, preservation of abdominal wall integrity, and enhanced patient satisfaction. Despite these advances, notable limitations persist. Prolonged operative times, high acquisition and maintenance costs, and the absence of tactile feedback remain critical barriers to widespread clinical adoption. Variability in surgeon experience and system-specific challenges, such as robotic arm collisions and limited instrument grip strength, further underscore the need for optimized training and system refinement. The reviewed studies were of moderate quality, primarily due to small sample sizes and lack of randomized comparative data. Future research should focus on cost-effectiveness analyses, long-term patient outcomes, and technical innovations, including biomimetic design, artificial intelligence, and soft robotics, to fully integrate robotics into plastic surgery. With interdisciplinary collaboration, robotic-assisted surgery holds the potential to redefine the standard of care in complex reconstructive interventions.

## Figures and Tables

**Table 1:** Summary of key outcomes from included studies.

Author and Year	Study Design	Type of Plastic Surgery	Robotic System Used	Advantages	Surgical Outcomes	Limitations and Challenges
Lai et al., 2019 [9]	Case series	Head and neck: free flap reconstruction for oropharyngeal cancer	da Vinci Surgical System	High-definition 3D visualization; tremor elimination	100% flap survival rate; precise suturing of vessels as small as 2.1 mm; no major complications reported	Lack of tactile feedback; long setup and operating times; high costs
Chung et al., 2015 [10]	Non-randomized experimental study	Breast reconstruction: RA latissimus dorsi muscle flap	da Vinci Surgical System	Flexible joints; motion scaling; tremor control	High patient satisfaction rates for aesthetics (mean scores: 9.2 to 9.9/10) and no donor site complications; improved aesthetics with hidden scars; docking time and operating time improved with experience	Difficulty dissecting anterior border due to simultaneous robot arm overlap and confined working space; large workspace required; increased time due to retractor adjustments; steep learning curve
Nadjmi 2016 [11]	Case series	Head and neck: cleft palate reconstruction	da Vinci Surgical System	3D endoscopic vision; tremor filtration; EndoWrist simulating hand-like movements	Improved dexterity and precision in intraoral suturing; shortened hospital stays (1 vs. 2.4 ± 1.3 days); normal swallowing restored on surgery day	Lack of tactile feedback; longer operative times (122 ± 8 min vs. 87 ± 6 min); high costs; steep learning curve
Arora et al., 2018 [12]	Case series	Head and neck: robotic resection of the tumor with free flap reconstruction	da Vinci Si Surgical System	Flexible robotic arms; high-definition 3D binocular vision; tremor elimination	100% flap survival; satisfactory functional and aesthetic outcomes; no postoperative complications; reduced surgical morbidity	Difficulty reconstructing flaps in narrow areas; limited access to recipient vessels; steep learning curve; longer docking/setup times
Lai et al., 2019 [13]	Case series	Breast reconstruction: RANSM with breast reconstruction	da Vinci Si Surgical Robot	3D imaging providing enhanced spatial precision; flexible robotic arms for fine dissection	87% of patients were graded as “excellent” for cosmetic outcomes; no total nipple-areolar complex necrosis was observed	Longer operative times; high costs
Ahn et al., 2019 [14]	Case series	Breast reconstruction: RANSM with immediate breast reconstruction	da Vinci Xi Surgical System	Flexible robotic arms; 3D magnified imaging	High patient satisfaction scores (BREAST-Q); invisible scars in most cases; no significant complications reported	High costs and initial long operative times; securing workspace was challenging
Moon et al., 2020 [15]	Case series	Chest reconstruction: RA latissimus dorsi muscle flap surgery for Poland Syndrome	da Vinci Surgical System	3D camera for a magnified view; robotic arms mimicking hand movements	High patient satisfaction for aesthetics and chest symmetry; inconspicuous scars achieved; no serious complications or flap loss reported	Lack of control group; high cost associated with robotic system purchase and maintenance; insufficient follow-up; prolonged operative time; potential bias in scar assessment
Jeon et al., 2021 [16]	Cohort study	Breast reconstruction: RA immediate prosthetic breast reconstruction	da Vinci Xi Surgical System	High-definition 3D imaging; articulated and flexible arms with precision motion; tremor reduction	Mean operative time: 194.7 min (oncology team), 80.8 min (plastic surgery team); minor complications (6% seroma, 6% superficial skin necrosis); small incisions (4.5 cm); enhanced visibility; precise tissue handling with minimal scarring	High costs; small patient cohort; short follow-up period; limited long-term outcomes or comparison with conventional techniques
Lin et al., 2021 [17]	Scientific report	Head and neck: genioplasty	Craniofacial-Plastic Surgical Robot (CPSR-I) system	Force feedback mechanism with automated drilling	Accurate osteotomy lines achieved high patient satisfaction without the need for additional surgeries	Heavy mechanical structure; insufficient navigation system for depth perception
Kanayama et al., 2021 [18]	Case series	Aesthetic: robotic recipient site preparation in hair transplantation	ARTAS Robotic System	Intelligent algorithms for hair identification; stereoscopic imaging	Efficient site creation with minimal complications; high patient and surgeon satisfaction ratings	Limited to the frontal scalp and requires preoperative hair trimming; familiarity affects speed initially
Hwang et al., 2022 [19]	Case series	Chest reconstruction: RA latissimus dorsi muscle flap surgery for Poland Syndrome	da Vinci SP Surgical System	Flexible robotic arms; 3D imaging; gas insufflation	Mean operative time: 449 min; no perioperative complications; superior scar aesthetics; enhanced operator ergonomics	Longer setup time for robotic docking compared to manual methods; high cost of robotic equipment; steep learning curve
Wittesaele et al., 2022 [20]	Case series	Breast reconstruction: RA DIEP flap	da Vinci Xi Surgical System (Multiport)	Tremor elimination, motion scaling; 3D imaging	No flap losses or conversions; reduced fascial disruption; successful flap survival with minimal complications	Longer operative times due to the learning curve, limited patient selection, high equipment costs
Weinzierl et al., 2023 [21]	Case series	Microsurgery: lymphatic microsurgery, including VLNT and LVA	Symani Surgical System with conventional or 3D exoscope	Motion scaling; tremor elimination; 3D depth perception	Precise anastomoses (mean time: 22.6 min); 25.2% limb volume reduction at 3 months; no complications	High cost; steep learning curve; fixed robotic angles required longer incisions in deep spaces
Tsai et al., 2023 [22]	Cohort study	Breast reconstruction: RA DIEP flap harvest	da Vinci Surgical System	Articulated robotic arms; high-definition 3D imaging	Reduced anterior rectus sheath incision length (RA 2.67 ± 1.13 cm vs. conventional 8.14 ± 1.69 cm); no flap loss; comparable pain scores; bilateral DIEP access achieved without robotic repositioning	RA procedure requires extra time (~100 min); costly disposables of approximately USD 3500; initial adaptation to post-placement technique
Aman et al., 2024 [23]	Cohort study	Hand surgery: RA peripheral nerve surgery	Symani Surgical System	Tremor reduction; precise nerve coaptations	Coaptation time averaged 23 min; 100% patency achieved; some complications like hematoma were reported	High cost; technical logistics; inferior grip strength of instruments; steep learning curve
Dastagir et al., 2024 [24]	Case series	Hand surgery: microsurgical anastomosis	Symani Surgical System	NanoWrist instruments; motion scaling; tremor reduction	Patency rate of 100%; significant ergonomic benefits; anastomosis time reduced by 30% with experience	Steep learning curve; equipment positioning; high cost of devices and training
Tolksdorf et al., 2024 [25]	Case series	Craniofacial surgery: free flap surgery with robotic-assisted and conventional anastomosis	Symani Surgical System with Orbeye exoscope for 3D magnification	NanoWrists; 7 degrees of freedom; motion scaling; tremor filtering	Anastomosis times longer with robotic methods; flap survival comparable to manual methods; minor complications reported	Long learning curve; technical issues (arm collisions, software errors); inadequate grip strength for thin sutures
Gorji et al., 2024 [26]	Case series	Breast, trauma, and head and neck surgery: free flap reconstruction	Symani Surgical System and RoboticScope	Motion scaling, telemetric control, orbital view adjustment	95.7% flap survival; ischemia time 100.6 min; end-to-side anastomoses performed efficiently	High costs; increased surgical time; intraoperative ventilation issues; robot setup complexity
Könnecker et al., 2024 [27]	Case series	Limb reconstruction: free tissue transfer with microsurgical anastomosis for extremity reconstruction	Symani Surgical System	Motion scaling, tremor filtering, NanoWrists, ergonomic telemanipulators	Successful flap survival; mean anastomosis time 33.2 min; no vascular complications	Small sample size; lack of comparative manual data; challenging grip strength for delicate movements
Farr et al., 2024 [28]	Case series	Breast reconstruction: RANSM	da Vinci SP Robotic Surgical System	Flexible 3D camera, robotic arms with improved motion range	Median operative time was 277 min; learning curve improvement was observed with time; minor complications observed	Limited to a single surgeon; insufficient generalizability; technical ease dependent on breast size
Oh et al., 2024 [29]	Case series	Breast surgery: RA capsulectomy	da Vinci SP Robotic Surgical System	Curved scissors; bipolar forceps; 3D magnified imaging	Enhanced precision in capsulectomy; clear visualization in confined spaces	Longer operative times than conventional methods; high costs associated with robotic setup
Kim et al., 2024 [30]	Cohort study	Breast reconstruction: immediate breast reconstruction following mastectomy (implant-based and autologous DIEP flap reconstruction)	da Vinci SP Robotic Surgical System	Articulated robotic arms; motion scaling; tremor reduction	Fewer complications (skin necrosis 2% robotic vs. 8% conventional); improved sexual well-being scores	Longer operative times for robotic procedures; high costs; steep learning curve for surgeons
Wong et al., 2024 [31]	Case-control study	Breast reconstruction: free flap reconstruction after endoscopic or robotic mastectomy	da Vinci Xi Surgical System	Flexible robotic arms mimicking human wrist movements; 3D imaging and tremor reduction	Scar placement was aesthetically superior in the robotic group; 70.7% of scars were hidden laterally compared to 70.7% in the conventional group	Aesthetic revision rates similar to conventional mastectomy; no direct aesthetic advantage with da Vinci Xi apart from scar position
von Reibnitz et al., 2024 [32]	Case series	Microsurgery: RA lymphatic reconstruction with LTT and/or LVA or LLA	Symani Surgical System	Motion scaling; tremor reduction; 7 degrees of freedom	Limb volume reduction of 1–10% (80 to 1250 mL); wound healing complications in 6.4% of cases; no need for surgical assistant	Lack of direct comparison with manual methods; inability to monitor long-term flap survival; single-center study
Jung et al., 2024 [33]	Cohort study	Breast reconstruction: RANSM with immediate breast reconstruction	da Vinci Xi Surgical System	Not specified	Significant improvements in operation times after 21 cases; mean docking time dropped from 8.2 to 5 min; minimal complications	Single-surgeon study; technical difficulties with the multiport systems, such as arm collisions and camera blind spots; limited tactile sensation was reported compared to conventional NSM
Lindenblatt et al., 2022 [34]	Case series	Lymphatic reconstruction	Symani Surgical System	Flexible robotic arms mimicking human wrist movements	100% patency; no complications	Small sample size; no follow up
Bier et al., 2023 [35]	Case series	Free flap reconstruction	Symani Surgical System	Motion scaling; tremor reduction; 7 degrees of freedom	Mean end-to-end arterial anastomosis time of 69 min; mean end-to-side arterial anastomosis time of 50 min; mean venous anastomosis time of 94 min; 4.3% flap loss rate; 21.7% revision rate.	Small sample size; no follow up
Besmens et al., 2023 [36]	Case series	Extremity (limb) free flap reconstruction	Symani Surgical System	Motion scaling; tremor reduction; 7 degrees of freedom	41.7 min mean end-to-end arterial anastomosis time; 29 min end-to-side arterial anastomosis; 22 min mean epineural coaptation time; 100% flap survival; 100% patency.	Small sample size; no follow up
Struebing et al., 2024 [37]	Case series	Free flap reconstruction, nerve reconstruction, lymphatic reconstruction	Symani Surgical System	Motion scaling; tremor reduction; 7 degrees of freedom	No statistically significant correlation between anastomosis time and case number; 31.0 ± 20.7 min end-to-end arterial anastomosis time; 37.4 ± 13.9 min end-to-side arterial anastomosis time; 23.8 ± 9.2 min venous anastomosis time; 28 ± 9.2 min LVA time; 16.5 ± 9.5 min epineural coaptation time; 2% partial flap loss rate; 2% revision rate.	No follow up
Grunherz et al. 2024 [38]	Case series	Central lymphatic reconstruction	Symani Surgical System	Motion scaling; tremor reduction; 7 degrees of freedom	Three-quarters of patients required further surgery or experienced symptoms within 7 months;1 patient asymptomatic at 11-month review	Small sample size; no follow up
Konneker et al., 2024 [39]	Case series	Free flap reconstruction	Symani Surgical System	Motion scaling; tremor reduction; 7 degrees of freedom	33.2 ± 5.8 min arterial anastomosis time; 100% flap survival; 12.5% revision rate	Small sample size; no follow up

RA = robot assisted, NSM = nipple-sparing mastectomy, RANSM = robot-assisted nipple-sparing mastectomy, DIEP = deep inferior epigastric perforator, VLNT = vascularized lymph node transfer, LVA = lymphaticovenous anastomosis, LTT = lymphatic tissue transfer, LLA = lympholymphatic anastomoses.

**Table 2:** Quality assessment of included studies.

Study	Focus	Study Design	Tool Used	Quality Assessment
Lai et al., 2019 [9]	Free flap for oropharyngeal cancer	Case series	CARE Checklist	Moderate quality
Chung et al., 2015 [10]	Breast reconstruction	Non-randomized experimental study	NOS	Moderate quality
Nadjmi 2016 [11]	Cleft palate reconstruction	Case series	CARE Checklist	Moderate quality
Arora et al., 2018 [12]	Tumor resection + free flap	Case series	CARE Checklist	Moderate quality
Lai et al., 2019 [13]	RANSM + breast reconstruction	Case series	CARE Checklist	Moderate quality
Ahn et al., 2019 [14]	RANSM + immediate breast reconstruction	Case series	CARE Checklist	Moderate quality
Moon et al., 2020 [15]	Chest reconstruction for Poland Syndrome	Case series	CARE Checklist	Moderate quality
Jeon et al., 2021 [16]	RA prosthetic breast reconstruction	Cohort study	NOS	Moderate quality
Lin et al., 2021 [17]	Genioplasty	Scientific report	Not applicable	Moderate quality
Kanayama et al., 2021 [18]	Hair transplantation	Case series	CARE Checklist	Moderate quality
Hwang et al., 2022 [19]	Chest reconstruction for Poland Syndrome	Case series	CARE Checklist	Moderate quality
Wittesaele et al., 2022 [20]	RA DIEP flap	Case series	CARE Checklist	Moderate quality
Weinzierl et al., 2023 [21]	Lymphatic microsurgery	Case series	CARE Checklist	Moderate quality
Tsai et al., 2023 [22]	RA DIEP flap harvest	Cohort study	NOS	Moderate quality
Aman et al., 2024 [23]	Peripheral nerve surgery	Cohort study	NOS	Moderate quality
Dastagir et al., 2024 [24]	Microsurgical anastomosis	Case series	CARE Checklist	Moderate quality
Tolksdorf et al., 2024 [25]	Free flap surgery (craniofacial)	Case series	CARE Checklist	Moderate quality
Gorji et al., 2024 [26]	Free flap reconstruction	Case series	CARE Checklist	Moderate quality
Könnecker et al., 2024 [27]	Limb reconstruction	Case series	CARE Checklist	Moderate quality
Farr et al., 2024 [28]	RANSM breast reconstruction	Case series	CARE Checklist	Moderate quality
Oh et al., 2024 [29]	Capsulectomy	Case series	CARE Checklist	Moderate quality
Kim et al., 2024 [30]	Immediate breast reconstruction	Cohort study	NOS	Moderate quality
Wong et al., 2024 [31]	Free flap after mastectomy	Case-control study	NOS	Moderate quality
von Reibnitz et al., 2024 [32]	Lymphatic reconstruction	Case series	CARE Checklist	Moderate quality
Jung et al., 2024 [33]	RANSM with breast reconstruction	Cohort study	NOS	Moderate quality
Lindenblatt et al., 2022 [34]	Lymphatic reconstruction	Case series	GRADE	Moderate quality
Bier et al., 2023 [35]	Free flap reconstruction	Case series	CARE Checklist	Moderate quality
Besmens et al., 2023 [36]	Extremity reconstruction	Case series	CARE Checklist	Moderate quality
Struebing et al., 2024 [37]	Flap, nerve, lymphatic recon.	Case series	CARE Checklist	Moderate quality
Grünherz et al., 2024 [38]	Central lymphatic reconstruction	Case series	CARE Checklist	Moderate quality
Konneker et al., 2024 [39]	Free flap reconstruction	Case series	CARE Checklist	Moderate quality

RA = robot assisted; NSM = nipple-sparing mastectomy; RANSM = robot-assisted nipple-sparing mastectomy; GRADE = Grading of Recommendations Assessment, Development and Evaluation; NOS = Newcastle–Ottawa Scale; CARE Checklist = Case Report (CARE) Checklist; SYRCLE = Systematic Review Centre for Laboratory Animal Experimentation Risk of Bias Tool.

**Table 3:** Advantages and disadvantages of common robotic systems.

Robotic System	Advantages	Disadvantages
Da Vinci Surgical System	High surgical precision with articulated robotic arms mimicking human wrist movements Enhanced 3D high-definition visualization and depth perception Motion scaling and tremor elimination Versatile application across breast reconstruction, head and neck surgery, microsurgery, and craniofacial procedures Facilitates minimally invasive approaches and improved aesthetic outcomes Well-established training programs and global accessibility	High cost of acquisition and maintenance Lack of haptic (tactile) feedback Steep learning curve, particularly for complex multi-quadrant procedures Requires significant operating room space Longer setup and docking times
Symani Surgical System	Designed specifically for microsurgical and supermicrosurgical procedures NanoWrists with 7 degrees of freedom allow precise movement in confined spaces Tremor filtration and motion scaling up to 20× enable manipulation of vessels <0.3 mm Compact design facilitates deep anatomical access Ergonomically optimized for reduced surgeon fatigue Demonstrated high success rates in lymphatic, nerve, and free flap procedures	Limited primarily to microsurgical applications High initial cost and limited global availability Requires team training and integration of exoscope or microscope for visualization Large working envelope required in complex reconstructions
ARTAS Robotic System	Intelligent algorithms for follicular unit extraction and graft placement Stereoscopic imaging enhances spatial accuracy Minimal trauma to surrounding follicles and tissue High consistency and reproducibility in graft placement High patient and surgeon satisfaction rates	Limited to hair transplantation procedures Requires preoperative hair trimming High cost and specialized training needed Not suitable for dense or scarred scalp tissue
Craniofacial Plastic Surgical Robot (CPSR-I)	Integrated force feedback improves tactile accuracy during osteotomy Precise bone cutting reduces revision rates Applicable in orthognathic and craniofacial contouring procedures Can be integrated with augmented reality for improved planning	Bulky design limits intraoperative flexibility Insufficient navigation system affects depth control Limited to craniofacial applications Compatibility issues with existing imaging and surgical platforms

## Data Availability

The data are available upon reasonable request to the corresponding author.
